# Internal Calibration Förster Resonance Energy Transfer Assay: A Real-Time Approach for Determining Protease Kinetics

**DOI:** 10.3390/s130404553

**Published:** 2013-04-08

**Authors:** Ling Jiang, Yan Liu, Yang Song, Amanda N. Saavedra, Songqin Pan, Wensheng Xiang, Jiayu Liao

**Affiliations:** 1 School of Life Science, Northeast Agricultural University, No. 59 Mucai Street, Xiangfang District, Harbin 150030, Heilongjiang, China; E-Mail: ling.jiang@ucr.edu; 2 Department of Bioengineering, Center for Bioengineering Research, Bourns College of Engineering, University of California at Riverside, 900 University Avenue, Riverside, CA 92521, USA; E-Mails: yliu024@ucr.edu (Y.L.); ysong004@ucr.edu (Y.S.); asaav001@ucr.edu (A.N.S.); 3 Institute for Integrative Genome Biology, University of California at Riverside, 900 University Avenue, Riverside, CA 92521, USA; E-Mail: songqin.pan@ucr.edu; 4 W. M. Keck Proteomics Laboratory, Institute for Integrative Genome Biology, University of California at Riverside, 900 University Avenue, Riverside, CA 92521, USA

**Keywords:** quantitative FRET analysis, internal calibration, one-sample assay, protease kinetics, SENP

## Abstract

Förster resonance energy transfer (FRET) technology has been widely used in biological and biomedical research. This powerful tool can elucidate protein interactions in either a dynamic or steady state. We recently developed a series of FRET-based technologies to determine protein interaction dissociation constant and for use in high-throughput screening assays of SUMOylation. SUMO (small ubiquitin-like modifier) is conjugated to substrates through an enzymatic cascade. This important posttranslational protein modification is critical for multiple biological processes. Sentrin/SUMO-specific proteases (SENPs) act as endopeptidases to process the pre-SUMO or as isopeptidases to deconjugate SUMO from its substrate. Here, we describe a novel quantitative FRET-based protease assay for determining the kinetics of SENP1. Our strategy is based on the quantitative analysis and differentiation of fluorescent emission signals at the FRET acceptor emission wavelengths. Those fluorescent emission signals consist of three components: the FRET signal and the fluorescent emissions of donor (CyPet) and acceptor (YPet). Unlike our previous method in which donor and acceptor direct emissions were excluded by standard curves, the three fluorescent emissions were determined quantitatively during the SENP digestion process from onesample. New mathematical algorithms were developed to determine digested substrate concentrations directly from the FRET signal and donor/acceptor direct emissions. The kinetic parameters, *k_cat_*, *K_M_*, and catalytic efficiency (*k_cat_*/*K_M_*) of SENP1 catalytic domain for pre-SUMO1/2/3 were derived. Importantly, the general principles of this new quantitative methodology of FRET-based protease kinetic determinations can be applied to other proteases in a robust and systems biology approach.

## 1. Introduction

SUMO (small ubiquitin-like modifier), a reversible posttranslational protein modifier, is essential in a variety of eukaryotic cellular events, including cell-cycle progression, transcriptional regulation, protein subcellular localizations, protein degradation and chromatin organization [1–5]. Mammalian cells usually express three SUMO paralogs (SUMO1, 2 and 3). A fourth paralog (SUMO4) was discovered in kidney cells and linked to a number of autoimmune diseases, but its biological role is unclear [6,7]. Like ubiquitination, SUMOylation is a dynamic and reversible process. SUMO conjugation occurs through a cascade of reactions that are performed by an activating enzyme (E1), a conjugating enzyme (E2) and, usually, a SUMO ligase (E3) [3,8,9]. The precursor of SUMO peptides needs to be processed by SENP (SUMO-specific protease) before the conjugation cascade [10,11]. The human SENP family of cysteine proteases has six true SENPs (*i.e.*, SENP1-3 and SENP5-7) [10,12,13].

The catalytic efficiency or specificity of an enzyme is best characterized by the ratio of the kinetic constants, *k_cat_*/*K_M_*. Several biochemical and fluorescent methods are commonly used to examine enzyme kinetics, including the western blotting, radioactive-labeled substrate, a fluorescent-labeled peptide substrate and a fluorescent protein–labeled substrate. All current methods have some limitations and drawbacks. For example, the maturation of pre-SUMO1, 2 and 3 by the catalytic domain of SENP1 was studied in solution and by sodium dodecyl sulfate-polyacrylamide gel electrophoresis (SDS-PAGE) [14,15]. The result showed SENP1's preference but cannot provide a detailed quantitative analysis due to the limitations of slow signal detection of the method. An organic fluorophore, 7-amino-4-carbamoylmethylcoumarin (ACC), was used to label a tetrapeptide substrate (QTGG). The ACC moiety was quenched in the intact substrate but became highly fluorescent upon cleavage by SENPs. Due to the distorted binding activity of small peptide substrate, the *k_cat_*/*K_M_* determined for SENP1 for the QTGG peptide was ∼300 M^−1^·s^−1^, which was up to two orders of magnitude lower than the natural substrates [15,16]. An assay-tagged 7-amino-4-methylcoumarin (AMC) with mature SUMOs was also developed. The values of *k_cat_*/*K_M_* wwere 2.4 × 10^6^ M^−1^·s^−1^ for SUMO1 and 5.6 × 10^5^ M^−1^·s^−1^ for SUMO2. These values are generally lower than traditionally considered enzyme kinetics numbers due to the reason noted above. In addition, this system cannot clearly differentiate the isopeptidase and endopeptidase activities of SENPs as there is no specific sequence of either SUMO tail or SUMO-specific substrate after the AMC moiety [17].

Time-resolved FRET assays have been used to characterize the deubiquitinating enzymes (DUBs) or SENPs. The FRET pair Eu-cryptate and allophycocyanin (APC) were tagged to anti-Myc and anti-FLAG antibodies, which interacted with Myc and FLAG on the N- and C-termini of pre-Nedd8, respectively [18]. Terbium (Tb) and YFP (yellow fluorescent protein), another FRET pair, were tagged on SUMO and anti-RanGAP, respectively, to study SUMOylation and SENP's deconjugation [19]. The same FRET pair was tagged onto the N- and C-termini of ubiquitin to study the DUB's processing by the time-resolved FRET (TR-FRET) [20]. However, these assays require additional steps for immune antibodies conjugation or chemical conjugation of thiol-reactive Tb chelate to ubiquitin-AC or other fluorophores. The low conjugation efficiency and denaturation during the conjugation process may lead to inaccurate results of quantitative analysis.

Recent studies focused on efforts to develop quantitative FRET techniques with differentiations of donor, acceptor and net FRET signal to determine molecular interaction events for the protein interaction dissociation constant *K_d_* by calibration curve [21] or direct estimation via direct “cross-talk” ratio measurements [22]. We developed a highly sensitive FRET-based protease assay for determining the kinetic parameters of SENP1 for pre-SUMO1s cleavage with a hybrid substrate, CyPet-preSUMO1-YPet [12]. The absolute FRET signal and direct emissions of the donor and acceptor were determined using two external standard curves (SC) of CyPet-SUMO1 and YPet, and CyPet-SUMO1, YPet and CyPet-preSUMO1-YPet, respectively. The absolute fluorescent signals were converted into protein concentrations by pre-established fluorescent protein standard curves. This method needs several steps to determine absolute FRET signal and convert it to protein concentrations, and during the conversions variability is introduced in each step of measurements.

Here, we report development of a novel quantitative FRET analytic method for determining SENP1 kinetics parameters from one-sample measurement with the “cross-talk” ratio of the donor and acceptor. The fluorescent emissions of the three components (*i.e.*, donor direct emission, acceptor direct emission and absolute FRET emission at acceptor emission wavelength) were monitored during the digestion of a CyPet-PreSUMO-YPet fusion substrate by SENP1. A new mathematical algorithm was developed to directly convert FRET signal to amount of digested substrate. The kinetics parameters, *K_M_*, *k_cat_* and *k_cat_*/*K_M_*, were then derived, and their values were compared with those obtained from the standard curve method. The results showed that *K_M_* and *k_cat_* from the real-time detection method are very close to those obtained from the standard curve method but with many fewer variations. These results suggest that this novel approach could be a very robust and reliable general method for protease kinetics determination.

## 2. Experimental

### 2.1. Plasmid Constructs

The open reading frames of the human SUMO and SENP1 genes were amplified by PCR, and the PCR products were cloned into the PCRII-TOPO vector (Life technologies, Carlsbad, CA, USA). After confirming the constructs by sequencing, the cDNAs encoding CyPet-(pre-SUMO1/2/3)-YPet, CyPet-SUMO1/2/3, YPet, and the catalytic domains of SENP1 were cloned into the pET28 (b) vector (Merck KGaA, Germany), engineered with an N-terminal 6xHistidine tag.

### 2.2. Protein Expression and Purification

*Escherichia coli* cells of strain BL21 (DE3) were transformed with pET28 vectors encoding CyPet-(pre-SUMO1/2/3)-YPet, CyPet-SUMO1/2/3, YPet, and the catalytic domains of SENP1. The transformed bacteria were grown in 2xYT medium to an optical density of 0.4–0.5 at 600 nm, by induction with 100 μM isopropyl-β-D-thiogalactoside for 16 hours at 25 °C. The 6xhistidine-tagged recombinant proteins were purified from bacterial lysates with nickel agarose affinity chromatography (Qiagen, Valencia, CA, USA) and eluted in 20 mM Tris-HCl, pH 7.4, 50 mM NaCl, 1 mM DTT. Protein purity was examined by SDS-PAGE, and concentrations of the purified proteins were determined by the Bradford assay (ThermoFisher Scientific, Waltham, MA, USA).

### 2.3. Self-Fluorescence Cross-Talk Ratio Determination

To determine the cross-talk ratio of CyPet and YPet's self-fluorescence, purified CyPet-SUMO1/2/3 and YPet were incubated individually in 37 °C in buffer containing 20 mM Tris-HCl, pH 7.4, 50 mM NaCl, 0.1% (v/v) Tween-20 and 1 mM DTT to a total volume of 80 μL at concentrations of 10, 20, 50, 100, 200, and 500 nM for 10 minutes and added to each well of a 384-well plate (Greiner, glass bottom).

Fluorescent emissions of CyPet at 475 and 530 nm were detected in a fluorescence multi-well plate reader (Flexstation II384, Molecular Devices, Sunnyvale, CA, USA) when excited at 414 nm to determine the cross-talk ratio *α*; fluorescent emissions of YPet at 530 nm were detected when excited at 414 and 475 nm to determine the cross-talk ratio *β*. Three samples were repeated for each concentration.

### 2.4. Establishing Standard Curves

CyPet-(pre-SUMO1/2/3)-YPet was incubated at 37 ° C in low-salt Tris buffer to a total volume of 80 μL and added to each well of a 96-well plate (Greiner, glass bottom). The emission signals at 475 nm were collected after excitation at 414 nm. The concentration of CyPet-(pre-SUMO1/2) was varied from 0.02 to 0.6 μM and of CyPet-(pre-SUMO3) from 0.4 to 3 μM.

CyPet-SUMO1/2/3 and YPet were incubated at 37 °C in a low-salt Tris buffer to a total volume of 80 μl with 1:1 molar ratio and added to each well of a 96-well plate (Greiner, glass bottom). The emission signals at 475 nm were collected after excitation at 414 nm. The concentration of CyPet-SUMO1/2 was varied from 0.02 to 0.2 μM, and of CyPet-(pre-SUMO3) varied from 0.05 to 0.5 μM.

### 2.5. Protease Kinetics Assay

FRET-based SUMO processing assays were conducted by measuring the emission intensity of CyPet at 475 nm and of YPet at 530 nm when excited at 414 nm in a fluorescence multi-well plate reader (Flexstation II^384^, Molecular Devices).

CyPet-(pre-SUMO1/2/3)-YPet was incubated with recombinant catalytic domain of SENP1 at 37 °C in buffer containing 20 mM Tris-HCl, pH 7.4, 50 mM NaCl, 0.1% (v/v) Tween-20 and 1 mM DTT to a total volume of 80 μL and added to each well of a 384-well plate (Greiner, glass bottom).

For the kinetics study of CyPet-(pre-SUMO1/2/3)-YPet processed by SENP1, substrate with different concentrations was digested with 0.15 and 4 nM SENP1 individually. Reactions were tested within original 5 min. One phase association model was used to fit the exponential increased reaction velocity. Data were analyzed by the developed method and plotted in GraphPad Prism V software to fit the Michaelis–Menten equation. Five samples were repeated in each concentration.

## 3. Results

### 3.1. Design of Internal Calibration Method to Determine Absolute FRET Signal

**Figure 1.**
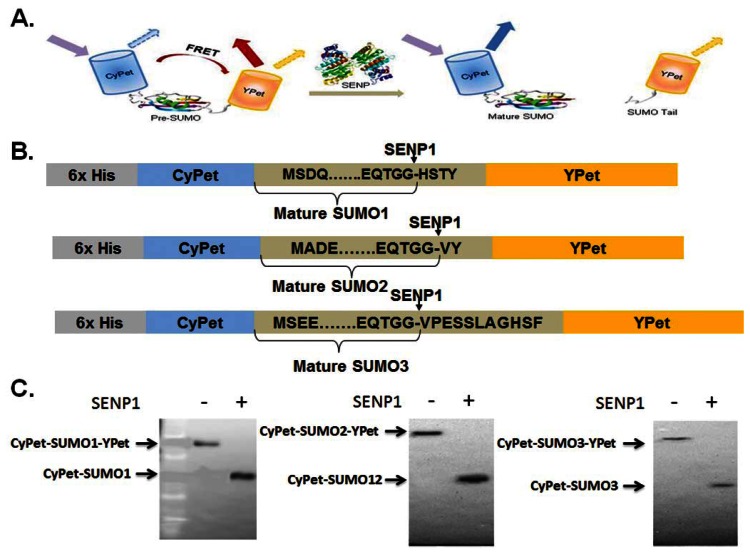
General design of the FRET-based SENP endopeptidase assay. (**A**) Schematic depicting the CyPet-(pre-SUMOs)-YPet substrate indicates the principle of FRET from CyPet (donor, excitation at 414 nm) to YPet (acceptor, emission measured at 530 nm). Once the protein is cleaved by SENP, the distance between the fluorescent proteins is increased beyond the FRET-sensitive distance, and thus, CyPet emission measured at 475 nm is increased, and the YPet FRET-induced emission is reduced. (**B**) Schematic of the 6His-CyPet-(pre-SUMO1/2/3)-YPet constructs. (**C**) Western-blots of CyPet-SUMO1/2/3-YPet before and after digestions by SENP1.

A FRET fusion substrate with a strong fluorescent signal for SENP1 endopeptidase activity measurement, CyPet-(pre-SUMO1/2/3)-YPet, was constructed. Digestion by SENP1 releases the products CyPet-SUMO1/2/3/ and the C-terminus of SUMO1/2/3-YPet and the FRET signal decreases (Figure 1), corresponding to the amount of digested substrate [23]. In our previous study of SENP1 and pre-SUMO1 digestion, we determined the change in the absolute FRET signal by subtracting the total fluorescence signal from YPet, the direct emission of the digested CyPet-SUMO1, and the emissions of remaining substrate. The emissions of digested YPet and CyPet-SUMO1 and the remaining substrate were determined using two external standard curves of an equal ratio of digested substrates, and digested substrates plus intact substrate, respectively [20]. This approach requires multiple steps to measure the emissions, and variation was introduced from the different assays. Therefore, we sought to determine donor and acceptor direct emissions by taking advantage of our recent development of “cross-talk” ratio methodology for quantitative FRET analysis [22,24].

**Figure 2.**
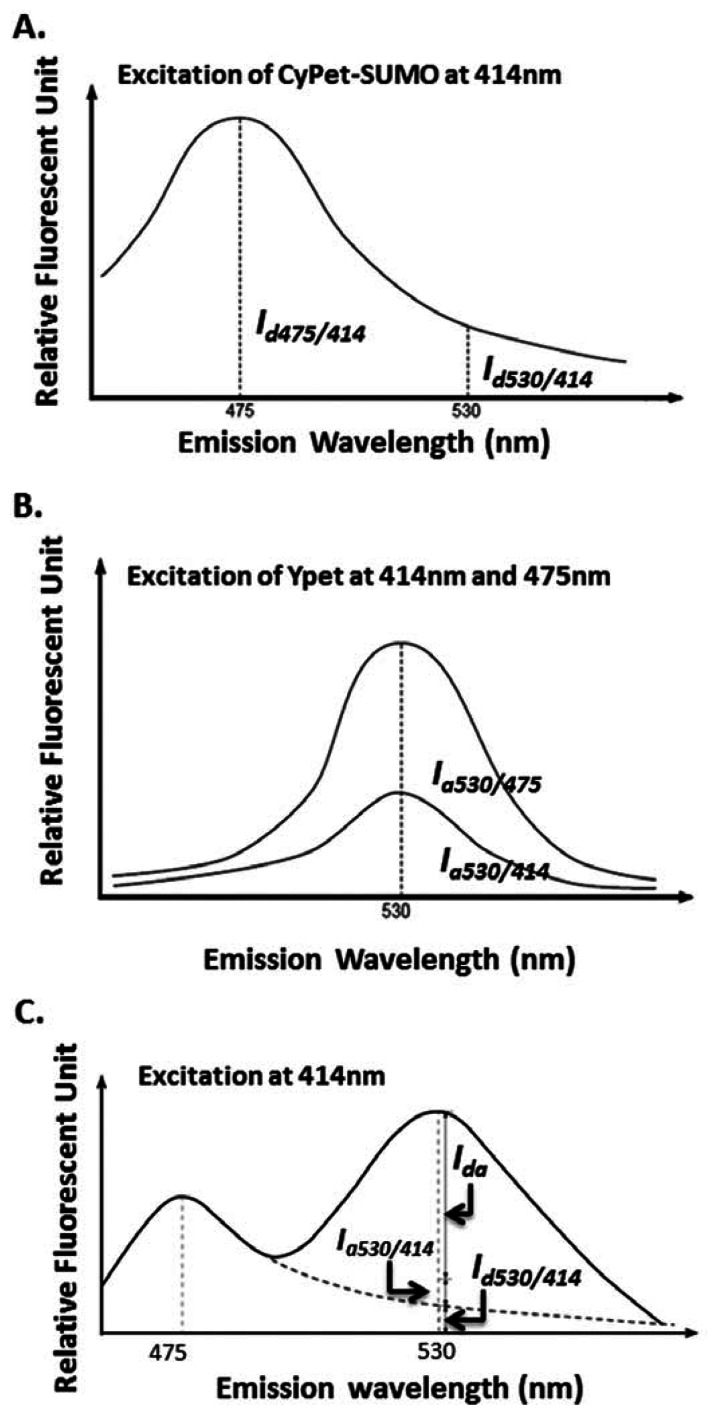
Spectrum analysis of FRET signal. (**A**). Spectrum analysis of direct emission of donor (CyPet). *I_d475_*_/_*_414_* is the fluorescent emission of CyPet-SUMO1/2/3 at 475 nm when excited at 414 nm; *I_d530_*_/_*_414_* is the fluorescent emission of CyPet-SUMO1/2/3 at 530 nm when excited at 414 nm. (**B**). Spectrum analysis of direct emission of acceptor (YPet). *I_a530_*_/_*_475_* is the fluorescent emission of YPet at 530 nm when excited at 475 nm; *I_a530_*_/_*_414_* is the fluorescent emission of YPet at 530 nm when excited at 414 nm. (**C**). Spectrum analysis of detected emission at 530 nm. Dissection of emission spectra from the engineered protein CyPet–(pre-SUMOs)–YPet when excited at 414 nm. *I_d_* is CyPet fluorescence at 475 nm when excited at 414 nm,*I_da_* is FRET-induced YPet emission at 530 nm when excited at 414 nm, and *I_a_* is direct YPet emission at 530 nm when excited at 475 nm.

The idea of a “cross-talk” ratio method for a fluorophore direct emission determination is to use two pre-determined ratios for donor and acceptor. The ratios depend on measurements at a different wavelength than the FRET signal emission wavelength (Figure 2(A)) or an emission of acceptor when excited at a wavelength different than FRET excitation wavelength (Figure 2(B)), respectively (Figure 2(C) and see Equations (1–4) below). Then, during the kinetics determination assay, one reaction sample was used to simultaneously determine three emissions, including the total fluorescence emission at the FRET signal emission wavelength, the emission of donor at its maximum emission wavelength of 475 nm when excited at a FRET excitation wavelength of 414 nm, and the emission of acceptor at its maximum emission wavelength of 535 nm when excited at its maximum excitation wavelength of 475 nm. To achieve the highest sensitivity, we chose the maximum emission or excitation wavelengths of donor or acceptor.

### 3.2. Cross-Talk Ratio Determinations of Cypet-Presumo1/2/3 and Ypet for Absolute FRET Signal Estimation from a One-Sample Measurement

The cross-talk ratio of CyPet's self-fluorescence (*α*) is the ratio of CyPet-PreSUMO1/2/3's emissions at 530 (*I_d530_*_/_*_414_, and I _d_* as fluorescent signal intensity of donor) to 475 nm (*I_d475_*_/_*_414_*) when excited at 414 nm (Figure 2(A)):

(1) $$\alpha =\frac{I_{d530/414}}{I_{d475/414}}$$

We determined the *α* values of CyPet-PreSUMO1/2/3 for each individual protein. The α values were 0.332, 0.278, and 0.265, respectively. The different values of *α* for each protein may be due to slight structural differences in the proteins.

The cross-talk ratio of YPet's self-fluorescence (*β*) is the ratio of YPet's emission at 530 nm when excited at 414 nm (*I_a530_*_/_*_414_* and *I_a_* as fluorescent signal intensity of acceptor) to the emission at 530 nm when excited at 475 nm (*I_a530_*_/_*_475_*) (Figure 2(B)):

(2) $$\beta =\frac{I_{a530/414}}{I_{a530/475}}$$

We determined the *β* value for YPet with purified YPet protein. The value determined for *β* was 0.026.

Therefore, we differentiated the total fluorescence signal at 530 nm when excited at 414 nm (*FL_530_*_/_*_414_*) as the contributions of three components: FRET-induced acceptor's emission (*FRET signal* or *Ida*), donor's direct emission(*I_d530_*_/_*_414_*) and acceptor's direct emission (*I_a530_*_/_*_414_*) (Figure 2(C)):

(3) $$FL_{530/414}=I_{da}+I_{d530/414}+I_{a530/414}$$

According to the definitions of *α* and *β*:

(4) $$FL_{530/414}=I_{da}+\alpha I_{d475/414}+\beta I_{a530/475}$$

where *I_d475_*_/_*_414_* is CyPet's emission at 475 nm when excited at 414 nm, and *I_a530_*_/_*_475_* is YPet's emission at 530 nm when excited at 475 nm.

After digestion by SENP1, the fluorescent signal at 530 nm decreased, and the fluorescent signal at 475 nm increased because of the disruption of FRET signal after substrate digestion. The remaining fluorescent emission at 530 nm (*FL′_530_*_/_*_414_*) could still be divided into a similar three components:

(5) $$FL_{530/414}^{\prime }=I_{da}^{\prime }+\alpha I_{d475/414}^{\prime }+\beta I_{a530/475}^{\prime }$$

where *I′_da_* is the remaining FRET-induced acceptor emission, *I′_d475_*_/_*_414_* is the fluorescent emission of CyPet, which consists of two parts: the undigested CyPet-(pre-SUMO1/2/3)-YPet and the digested CyPet-SUMO1/2/3, and *I′_a530_*_/_*_475_* is the fluorescent emission of YPet, which is constant whether substrate is digested or not.

### 3.3. Determine Digested Substrate Concentration from the FRET Signal Changes, Which Are Determined by Internal Calibration Method

The amount of digested substrate is correlated with the decrease of absolute FRET signal. Therefore, after treatment with SENP1, the remaining FRET-induced acceptor's emission $\left(I_{da}^{\prime }\right)$ was:

(6) $$I_{da}^{\prime }=\frac{c-x}{c}\times I_{da}=\frac{c-x}{c}\times (FL_{530/414}-\alpha I_{d475/414}-\beta I_{a530/475)}$$

where *C* is the original concentration of substrate CyPet-(pre-SUMO1/2/3)-YPet(μM), and *x* is the concentration of digested CyPet-(pre-SUMO1/2/3)-YPet (μM).

In this newly developed internal calibration (IC) method, *I′_d475_*_/_*_414_* can be directly detected during the experiment. Therefore, the fluorescent signal detected at 530 nm when excited at 414 nm can be expressed as:

(7) $$FL_{530/414}^{\prime }=\frac{c-x}{c}\times (FL_{530/414}-\alpha I_{d475/414}-\beta I_{a530/475})+\alpha I_{d475/414}^{\prime }+\beta I_{a530/475}$$

During the experiment, $FL_{530/414}^{\prime }$ and $I_{d475/414}^{\prime }$were directly measured by the instrument, Flexstation II^386^, whereas, *FL*_530/414_, *I_d_*_475/414_,*I_a_*_530/475_, *α* and *β* were predetermined before the experiment. So, the two fluorescence signals for determining the amount of the digested substrate are determined during the experiment in real time.

**Figure A1.**
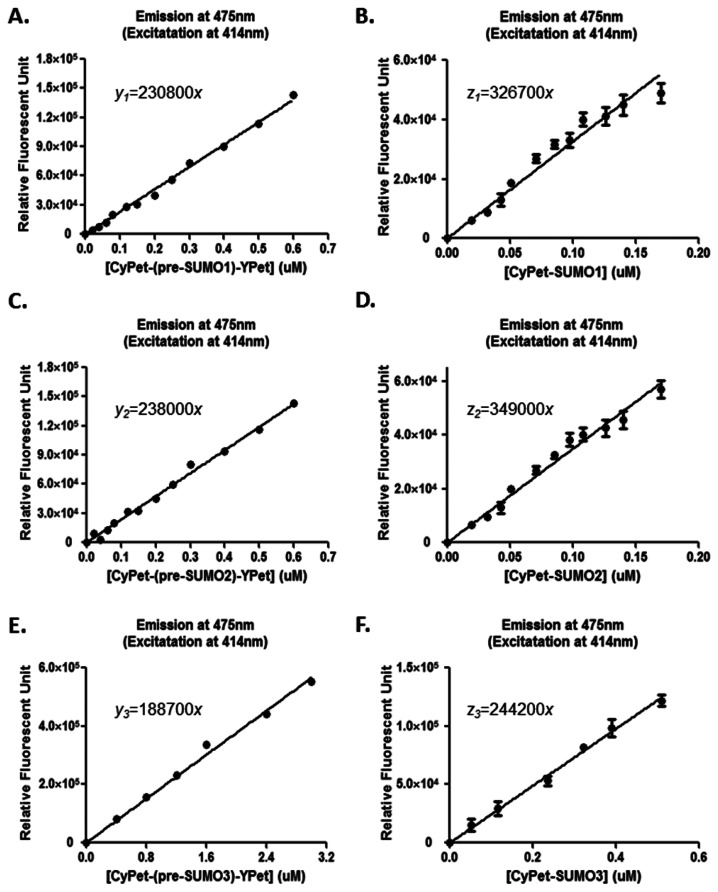
Standard curves of fusion substrates and digested products. (**A**). CyPet-(pre-SUMO1)-Ypet. (**B**). CyPet-SUMO1 with YPet (1:1 molar ratio). (**C**). CyPet-(pre-SUMO2)-Ypet. (**D**). CyPet-SUMO2 with YPet (1:1 molar ratio). (**E**). CyPet-(pre-SUMO3)-Ypet. (**F**). CyPet-SUMO3 with YPet (1:1 molar ratio).

In the previous method [23], standard curves were established to correlate the undigested and digested substrate concentrations to fluorescent signals (Appendix Figure A1). The direct emission of CyPet was determined from both the digested CyPet-SUMO1/2/3 (*I′_d-cs_*) and the remaining CyPet-(pre-SUMO1/2/3)-YPet (*I′_d-csy_*) substrates:

(8) $$I_{d475/414}^{\prime }=I_{d475/414-csy}^{\prime }+I_{d475/414-cs}^{\prime }$$

According to the established standard curves:

(9) $$I_{d475/414-csy}=y=k\;(C-x)$$

(10) $$I_{d475/414-cs}=z=jx$$

where *I_d475_*_/_*_414-csy_* (or *y* which is fluorescence signal intensity of undigested substrate in the standard curve) is the emission intensity of CyPet-(pre-SUMO1/2/3)-YPet at 475 nm when excited at 414 nm, *k* is slope of the standard curve for the emission intensity (*I_d-csy_*) to the concentration of undigested CyPet-(pre-SUMO1/2/3)-YPet (μM) (*k* is 230,800 for SUMO1; 238,000 for SUMO2 and 188,700 for SUMO3 fusion proteins), *I_d475_*_/_*_414-cs_* (or *z* which is the fluorescence signal intensity of the digested substrate in standard curve) is the emission intensity of CyPet-SUMO1/2/3 at 475 nm when excited at 414 nm, and *j* is the slope of the standard curve for the emission intensity (*I_d-cs_*) to concentration of digested CyPet-SUMO1/2/3 (μM) (*j* is 326,700 for SUMO1; 349,000 for SUMO2; and 244,200 for SUMO3 fused with CyPet).

**Figure 3.**
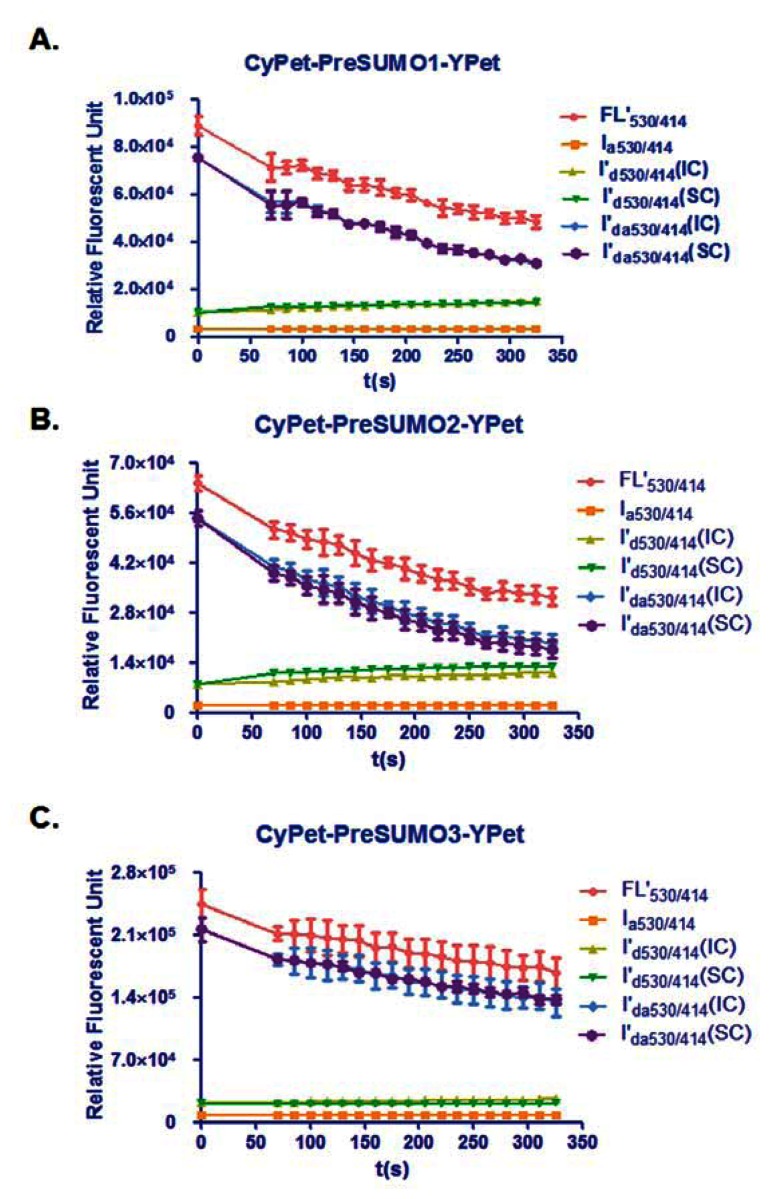
Time course of fluorescent signal changes of SUMO1/2/3 substrates during SENP1 digestion. **(A**) Fluorescent signal changes of CyPet-PreSUMO1-Ypet digested by SENP1. FL′_530/414_ is total fluorescent emission of digestion sample when excited at 414 nm. I_a530/414_ is the emission of YPet at 530 nm when excited at 414 nm. I′_d530/414_ (IC) is emission of CyPet at 530 nm when excited at 414 nm by the internal calibration method. I′_d530/414_ (SC) is emission of CyPet at 530 nm when excited at 414 nm by the standard curve assay. I′_da530/414_ (IC) is the FRET emission of YPet at 530 nm from CyPet when excited at 414 nm by the real-time method. I′_da530/414_ (SC) is the FRET emission of YPet at 530 nm from CyPet when excited at 414 nm by the standard curve method. (**B**) Fluorescent signal changes of CyPet-PreSUMO2-Ypet digested by SENP1. All the curves are labeled as same as in (A) except all the signals come from the substrate of CyPet-PreSUMO2-Ypet. (**C**) Fluorescent signal changes of CyPet-PreSUMO3-Ypet digested by SENP1. All the curves are labeled as same as in (A) except all the signals come from the substrate of CyPet-PreSUMO2-Ypet.

In this study, the total fluorescent emission detected at 530 nm, CyPet and YPet direct emission, as well as the FRET-induced YPet's emission, was analyzed by the internal calibration method and directly compared with those determined by the standard curve method for the concentration of CyPet-(pre-SUMO1)-YPet at 0.15 μM, CyPet-(pre-SUMO2)-YPet at 0.15 μM, and CyPet-(pre-SUMO3)-YPet at 0.4 μM (Figure 3). In all the experiments, the fluorescent signal changes from the internal calibration method were very similar to these determined by the standard curve method, but with significantly less variation and less variation in the final kinetics determinations (see Table 1 below). In addition, the internal calibration method eliminates the need of the standard curves and reduces significant amount experimental procedure.

### 3.4. Initial Velocity Determination of SENP1 with Pre-SUMO1/2/3 Substrates

**Figure 4.**
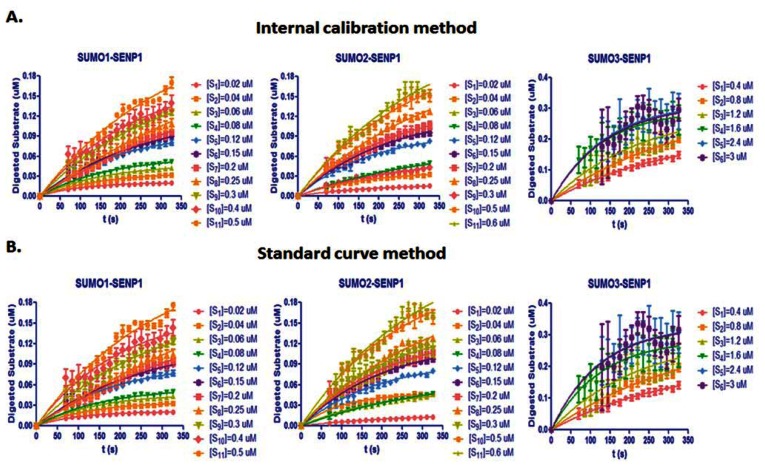
Substrate digested velocities of PreSUMO1,2,3 by SENP1. (**A**) Substrate digestion velocity of PreSUMO1 (left), PreSUMO2 (middle), and PreSUMO3 (right) by SENP1 in a real-time FRET assay. (**B**) Substrate digestion velocity of PreSUMO1 (left), PreSUMO2 (middle), and PreSUMO3 (right) by SENP1 by the standard curve FRET assay.

From Equation (7), the digested substrate at different time points can be calculated after the absolute FRET signal changes were determined by the real-time internal calibration method. The amounts of digested substrate, CyPet-(pre-SUMO1/2/3)-YPet, were plotted against the digestion time (Figure 4(A)). The amounts of digested substrates were also determined by the standard curve method (Figure 4(B)). These two methods show similar digestion curves, but with less variation from the internal calibration method. Different amounts of the fluorescent substrates, CyPet-(pre-SUMO1)-YPet (0.02–0.5 μM), CyPet-(pre-SUMO2)-YPet (0.02–0.6 μM), and CyPet-(pre-SUMO3)-YPet (0.4–3.0 μM), were incubated with 0.15 nM (for SENP1/2), and 4 nM (for SENP3) catalytic domain of SENP1, respectively. The emissions at 530 nm and 475 nm, when excited at 414 nm, were determined. A greater substrate concentration for pre-SUMO3 was used because the digestion activity of SENP1 for pre-SUMO3 is much less efficient, and signal changes were very small. This was confirmed later in *K_cat_*/*K_M_* determination. The digested substrates, *x*, were then calculated by the both internal calibration (Figure 4(A)) and the standard curve methods (Figure 4(B)).

The initial velocity (*V_o_*) of CyPet-(pre-SUMO1/2/3)-YPet's maturation by SENP1 was determined as described [23]. Briefly, to determine the reaction velocity of SENP1, the reaction rate (*v*) was correlated to the change in the amount of substrate (*S*):

(11) $$v=-\frac{d\;\left[S\right]}{dt}=\frac{d\;\left[P\right]}{dt}$$

As the digested substrate (or product) concentration increases exponentially from 0 when t = 0, to [*S*]_0_ at infinite time:

(12) $$\left[P]={\left[S\right]}_{0}\;(1-e^{-kt}\right)$$

Accordingly, the original velocity (*v_0_*) of CyPet-(pre-SUMO1)-YPet's maturation by SENP1 is:

(13) $$V_{0}=d\;[p]/dt_{t=0}=k\;{\left[s\right]}_{0}$$

The results of initial velocity at different concentrations of substrates determined by the two methods are reported in Appendix Table A1. These results also suggest that SENP1 prefers the pre-SUMO1/2 substrates over the pre-SUMO3 substrate. The results displayed a good substrate dose-dependent relationship and showed the small differences from the above two methods.

### 3.5. Enzyme Kinetics Determination by Michaelis-Menten Analysis

The catalytic specificity and efficiency of an enzyme for a specific substrate are best defined by the ratio of the kinetic constants, *k_cat_*/*K_M_*. This ratio is generally used to compare the efficiencies of different enzymes towards one substrate or different substrates catalyzed by one enzyme.

**Figure 5.**
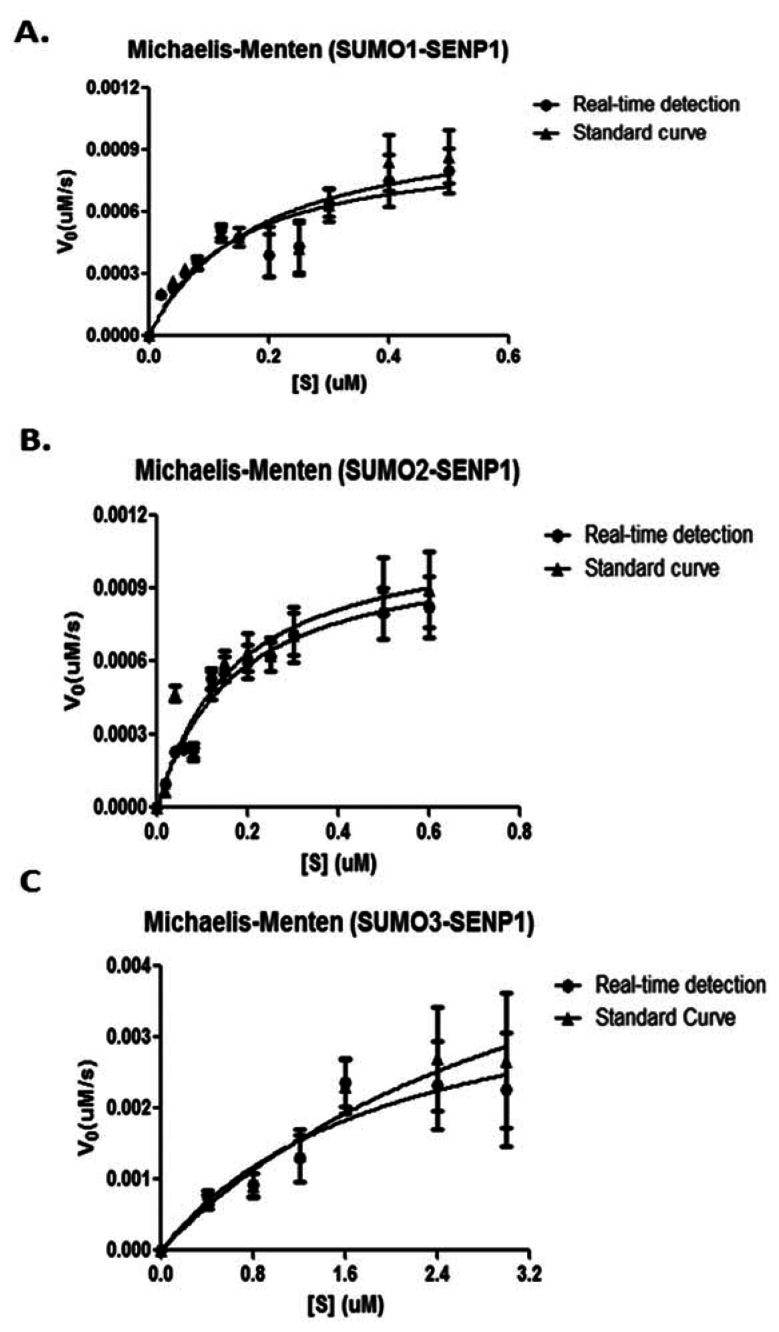
Michaelis-Menten plots of PreSUMO digestions by SENP1. (**A**). Michaelis-Menten plots of pre-SUMO1 digestion by SENP1 by either a real-time or standard curve method. (**B**). Michaelis-Menten plots of pre-SUMO1 digestion by SENP1 by either a real-time or standard curve method. (**C**). Michaelis-Menten plots of pre-SUMO1 digestion by SENP1 by either a real-time or standard curve method.

The *K_M_* and *V_max_* values can be obtained from the Michaelis-Menten equation by plotting the initial velocities of SENP1 digestion versus the corresponding concentrations of different substrates. The obtained initial velocities in the Appendix Table A1 were plotted in Michaelis-Menten models for pre-SUMO1/2/3, respectively (Figure 5). The derived values of *K_M_*, *k_cat_* and *k_cat_*/*K_M_* ratio were listed in the Table 1.

Compared to a previous study of SENP1's activity by ratiometric method analyzed FRET-based protease assay [25], we obtained similar values for the ratios of *k_cat_*/*K_M_* of pre-SUMO1 (4.41 × 10^7^*vs.* 3.92 × 10^7^ M^−1^·s^−1^), pre-SUMO2 (4.28 × 10^7^*vs.* 4.60 × 10^7^ M^−1^·s^−1^) and pre-SUMO3 (5.2 × 10^5^*vs.* 5.95 × 10^5^ M^−1^·s^−1^). The values of *K_M_*, *k_cat_* and *k_cat_*/*K_M_* ratio for pre-SUMO1 and pre-SUMO2's maturation by SENP1 were very similar. In contrast, the catalytic activity of SENP1 for pre-SUMO3 is much less than the pre-SUMO1 and pre-SUMO2.

## 4. Discussion

Here, we report a novel quantitative FRET method for determining protease SENP1 kinetics from one-sample measurement in real time for a SUMO systems biology application. In this approach, the absolute FRET signal that is correlated to the digested substrate was continuously determined during the SENP1 digestions of CyPet-pre-SUMO1/2/3-YPet substrate. New mathematic algorithms were developed to differentiate absolute FRET signal from the direct emissions of donor and acceptor at 530 nm, and derive digested substrate from the absolute FRET signal in real time. The direct emission of CyPet at 530 nm, which can be divided into two parts (*i.e.*, undigested CyPet-(pre-SUMOs)-YPet and digested CyPet-SUMOs), was directly obtained from the cross-talk ratio, α. The CyPet emission at 475 nm was determined during digestion in real time, and the values were used to determine the CyPet direct emission at 530 nm. The YPet direct emission at 530 nm was determined by the product of its emission at 530 nm when excited at 475 nm and the cross-talk ratio, β.

In our previous study, the kinetics of pre-SUMO1's maturation by SENP1 was characterized by differentiating and quantifying absolute fluorescent signals contributed by the donor and acceptor emissions and the FRET signal that were then converted into protein concentrations by pre-established fluorescent protein standard curves. The calculated *K_M_*, *k_cat_* and *k_cat_*/*K_M_* ratios of these two methods were compared in Table 2. The *k_cat_*/*K_M_* ratios were similar, but the standard curve method produced greater variation, which came from the errors of standard curves themselves and multiple spectrometer readings. Slopes of standard curves were derived from the linear fitting of fluorescent readings to the protein concentrations. Errors of the slopes cannot be avoided as they result from variations in each protein sample preparation. Nevertheless, the fluorescent emissions for the standard curves were varied slightly from different batches of the recombinant proteins. In addition, the standard curves are needed for each experiment, which is also very tedious. On the other hand, according to a review of different quantitative FRET analytic methods [26], more accurate and robust results can be obtained by observations from multiple channels instead of only one. In this improved approach, real-time fluorescent signals from both 475 and 530 nm were determined, but the previous standard curve method only analyzes the fluorescent signal from 530 nm. Although the derived values for the kinetic parameters were similar, the higher variations form the standard error method indicates that the improved real-time detection method simplifies the measurements by skipping the need of standard curve establishment, and therefore, this method provided more accurate and consistent results.

Pre-SUMO's maturation by SENP1 has been studied by other methods, such as protein gel-based methods and a ratiometric-based FRET assay [14,15,25]. For example, the previous results from gel-based studies showed that SENP1 digests the pre-SUMO1 more efficiently than the pre-SUMO2 and, in turn, more than the pre-SUMO3 [27]. However, the pre-SUMO2 used in the study had an additional nine amino acids after SUMO2's VY tail, and the natural pre-SUMO2 cannot be cleaved in this approach [19]. However, because the gel-based technology is not very quantitative and slow tracking process, the enzymatic kinetics of the maturation process cannot be determined. According to our results, the catalytic activities of SENP1 for pre-SUMO1 and pre-SUMO2 are almost the same in both binding and catalysis steps, and these catalytic activities are much more efficient than for the pre-SUMO3, which is comparable to the previous studies as pre-SUMO1>pre-SUMO3 [20].

Fluorescent proteins can be genetically tagged to interested proteins. CFP (cyan fluorescent protein) and YFP were used as a FRET pair to study the SENP1's endopeptidase activities [25,28]. The *k_cat_*/*K_M_* determined from the assay was 3.8 × 10^7^ M^−1^·s^−1^ for SUMO1 and 5.95 × 10^5^ M^−1^·s^−1^ for SUMO3. However, the above FRET-based protease assays used the ratio of acceptor's emission to donor's emission (under the excitation of donor) as the quantitative parameter to characterize the FRET signals without considering the self-fluorescence from donors and acceptors. Although the numerical values of kinetics are similar to ours, this method resulted from an inaccurate FRET signal analysis may lead to wrong conclusions if different FRET pairs are used [26,29].

In summary, we developed a new theoretical and experimental procedure for protease kinetics parameter determination based on quantitative FRET assay of high-sensitive hybrid fluorescent sensor. Except the two pre-determined “cross-talk” ratios of donor and acceptor that should be universal for a specific substrate, all the measurements are carried out from one reaction sample. This approach will significantly shorten experimental procedure and also reduce experimental variations because of minimum experimental steps. This methodology can be applied for any FRET pairs and protease in general.

## Figures and Tables

**Table 1.** *k_cat_*/*K_M_* of SENP1 for SUMO1,2,3 determined by the real-time detection and standard curve analysis.

	**FRET signal Analysis method**	***K****_m_***(μM)**	***K****_cat_***(S^−1^)**	***K****_cat_***/*K****_m_***(M^−1^**·**S^−1^)**
**SUMO1**	Real-time detection	0.14 ± 0.05	6.14 ± 0.89	(4.41 ± 1.74) × 10^7^
	Standard curve	0.18 ± 0.08	7.07 ± 1.33	(3.92 ± 1.85) × 10^7^

**SUMO2**	Real-time detection	0.17 ± 0.003	7.17 ± 0.54	(4.28 ± 0.83) × 10^7^
	Standard curve	0.17 ± 0.01	7.66 ± 1.04	(4.60 ± 1.66) × 10^7^

**SUMO3**	Real-time detection	1.99 ± 1.25	1.03 ± 0.33	(5.17 ± 3.65) × 10^5^
	Standard curve	3.75 ± 2.22	1.61 ± 0.62	(4.31 ± 3.04) × 10^5^

## Appendix

**Table A1.** Initial velocity determinations of pre-SUMO1, 2, and 3 by real-time detection and standard curve analysis.

**Table A1-1.** Initial velocity determinations of SENP1 for SUMO1.		

**[S[(μM)**	***Vo*(μM/s)**	

	**Real-Time Detection**	**Standard Curve**
0.02	0.00020 ± 0.000010	0.00021 ± 0.000010
0.04	0.00023 ± 0.000014	0.00026 ± 0.000016
0.06	0.00030 ± 0.000017	0.00033 ± 0.000018
0.08	0.00035 ± 0.000029	0.00035 ± 0.000028
0.12	0.00051 ± 0.000033	0.00050 ± 0.000037
0.15	0.00048 ± 0.000044	0.00048 ± 0.000044
0.2	0.00039 ± 0.000010	0.00041 ± 0.000012
0.25	0.00044 ± 0.000013	0.00042 ± 0.000013
0.30	0.00064 ± 0.000069	0.00063 ± 0.000079
0.50	0.00075 ± 0.000013	0.00084 ± 0.000013
0.60	0.00080 ± 0.000011	0.00087 ± 0.000013

**Table A1-2.** Initial velocity determinations of SENP1 for SUMO1		

**[S[(μM)**	***Vo*(μM/s)**	

	**Real-time detection**	**Standard curve**
0.02	0.000096 ± 0.000007	0.000065 ± 0.000008
0.04	0.00023 ± 0.000022	0.00047 ± 0.000031
0.06	0.00024 ± 0.000020	0.00026 ± 0.000023
0.08	0.00024 ± 0.000031	0.00022 ± 0.000028
0.12	0.00053 ± 0.000043	0.00050 ± 0.000056
0.15	0.00057 ± 0.000049	0.00060 ± 0.000048
0.2	0.00060 ± 0.000068	0.00064 ± 0.000081
0.25	0.00062 ± 0.000059	0.00063 ± 0.000068
0.3	0.00071 ± 0.000088	0.00071 ± 0.000011
0.5	0.00080 ± 0.000011	0.00091 ± 0.000012
0.6	0.00082 ± 0.000013	0.00090 ± 0.000016

**Table A1-3.** Initial velocity determinations of SENP1 for SUMO1.		

**[S[(μM)**	***Vo*(μM/s)**	

	**Real-time detection**	**Standard curve**
0.4	0.00075 ± 0.000094	0.00068 ± 0.000010
0.8	0.00093 ± 0.000016	0.00091 ± 0.000017
1.2	0.0013 ± 0.00033	0.00013 ± 0.000037
1.6	0.0024 ± 0.00034	0.00027 ± 0.000073
2.4	0.0023 ± 0.00062	0.00027 ± 0.000095
3.0	0.0023 ± 0.00080	0.00027 ± 0.00016

## Abbreviations

Ubl: Ubiquitin-like protein
SUMO: Small ubiquitin-like modifier
SENP: SUMO/Sentrin specific protease
SDS-PAGE: Sodium dodecyl sulfate-polyacrylamide gel electrophoresis
ACC: 7-Amino-4-carbamoylmetylcoumarin
AMC: 7-Amino-4-methylcoumarin
FRET: Förester resonance energy transfer
DUB: Deubiquitinating enzyme
CFP: Cyan fluorescent protein
YFP: Yellow fluorescent protein
DTT: Dithiothreitol
